# Use video comprehension technology to diagnose ultrasound pneumothorax like a doctor would

**DOI:** 10.3389/fphys.2025.1530808

**Published:** 2025-05-27

**Authors:** Xiaoyong Qiang, Qiang Wang, Guanjun Liu, Limei Song, Weibin Zhou, Ming Yu, Hang Wu

**Affiliations:** ^1^ College of Electronic Information and Automation, Tianjin University of Science and Technology, Tianjin, China; ^2^ Systems Engineering Institute, Academy of Military Sciences, People’s Liberation Army, Tianjin, China; ^3^ School of Artificial Intelligence, Nankai University, Tianjin, China

**Keywords:** pneumothorax, lung ultrasound, medical imaging, intelligent diagnostics, deep learning, video understanding

## Abstract

**Introduction:**

Emergency rescue scenes and pre-hospital emergency stages commonly encounter trauma victims. Life-saving measures must be taken at the scene if a trauma patient has pneumothorax; if the patient is not evaluated and diagnosed right away, their life may be in jeopardy. Ultrasound, which has the advantages of being non-invasive, non-radioactive, portable, rapid, and repeatable, can be used to diagnose pneumothorax. However, those who interpret ultrasound images must undergo extensive, specialized, and rigorous training. Deep learning technology allows for the intelligent diagnosis of ultrasound images, allowing general healthcare professionals to quickly and with minimal training diagnose pneumothorax in lung ultrasound patients.

**Methods:**

Previous studies focused primarily on the lung-sliding characteristics of M-mode images, neglecting other key features in lung ultrasonography pneumothorax, and used similar technological techniques. Our study team used video understanding technology for medical ultrasound imaging diagnostics, training the TSM video understanding model on the ResNet50 network with 657 clips and testing the model with untrained 164 lung ultrasound clips.

**Results:**

The model’s sensitivity was 99.21%, specificity was 89.19%, and average accuracy was 96.95%. The F1 score was 0.929, and the AUC was 0.97.

**Discussion:**

This study is the first to apply video understanding models to the multi-feature fusion diagnosis of pneumothorax, demonstrating the feasibility of using video understanding technology in medical image diagnosis.

## 1 Introduction

Trauma is one of the most prevalent reasons for emergency room visits, as well as a leading cause of death (Qian and Zhang, 2019). Pneumothorax (PTX), or lung collapse, is a life-threatening respiratory emergency that can occur in trauma patients as well as individuals with acute and chronic diseases (Duclos et al., 2019; Weissman and Agrawal, 2021) and must be treated immediately (Lichtenstein, 2015). Ultrasonography has the virtue of being quick, repeatable, and noninvasive, and it has become an essential injury detection tool in the emergency room (Valenzuela et al., 2020). PTX can be recognized early and promptly using the E-FAST (Extended Focused Assessment with Sonography in Trauma) technique and treated symptomatically (Hefny et al., 2019). Despite the fact that lung ultrasound (LUS) has higher diagnostic accuracy than chest X-ray for PTX (Nagarsheth and Kurek, 2011; Alrajhi et al., 2012) and offers additional benefits, a lack of training opportunities and clinician-centered workflows continue to impede widespread use (Brady et al., 2021).

AI-powered ultrasound interpretation eliminates training and workflow bottlenecks, allowing non-specialists to use the technology to provide fast, portable imaging on a large scale (VanBerlo et al., 2022). This study is built on this objective, and it uses deep learning networks to learn and recognize pneumothorax features in ultrasound images, as well as to achieve automatic recognition and diagnosis of pneumothorax ultrasound images. An auxiliary tool is supplied to healthcare workers to ease the process of interpreting ultrasound images, shorten the learning cycle, and lower the barrier to using ultrasound equipment. Our goal is for even general healthcare workers with basic training to be able to operate ultrasound equipment proficiently for pneumothorax diagnosis and obtain diagnostic accuracy comparable to that of specialized imaging physicians. This will not only help improve accessibility and efficiency of healthcare but will also improve patient outcomes by providing rapid and reliable diagnostic information in emergencies.

Because PTX assessment is a critical component in the identification of complex life-threatening patient situations such as trauma (Blaivas et al., 2005), cardiac arrest (Hernandez et al., 2008), and respiratory distress (Wallbridge et al., 2018), detecting the existence of PTX is a crucial component of lung ultrasonography. At the time of writing, there is a limited amount of relevant literature on the diagnosis of pneumothorax by AI ultrasound, and no publicly available datasets on ultrasound pneumothorax have been collected. The majority of studies on PTX are based on publicly available CT chest radiograph datasets or X-ray chest radiograph datasets, with a few based on private ultrasound datasets (animal experimental data, simulated material data, real-life ultrasound data).

Some recognized research claims to have produced relatively good results for automated PTX detection in animal ultrasonography investigations (Mehanian et al., 2019; Kulhare et al., 2018; Summers et al., 2017; Lindsey et al., 2019), but human accuracy is unknown. Boice et al. (2022) used synthetic gelatin models cast in 3D-printed rib molds and a simulated lung. M-mode ultrasound pneumothorax simulation images were obtained and used to train the pneumothorax detection model, which was tested using animal ultrasound data and ended up with an overall accuracy of 93.6%. Jaščur et al. (2021) employed a convolutional neural network (CNN) model on a restricted dataset with 82% sensitivity to missing lung sliding. VanBerlo et al. (2022) used a large labeled LUS dataset from two academic institutions to classify images after converting B-mode movies to M-mode images, and the model had a sensitivity of 93.5% to lung sliding, specificity of 87.3%, and AUC of 0.973. Kim et al. (2023) proposed an AI-assisted pneumothorax diagnosis framework that simulates clinical workflows through sequential steps, including pleural line localization under B-mode ultrasound, B-to-M-mode image reconstruction, and lung sliding detection. Utilizing lightweight models (<3 million parameters), they achieved pleural line quality assessment (Dice coefficient: 89%) and sliding classification individually, with single-model AUCs exceeding 95% and an overall workflow AUC of 89%.

However, Lichtenstein and Menu (Lichtenstein and Menu, 1995) clearly stated that although the disappearance of lung sliding is observed in 100% of PTX cases, the disappearance of lung sliding may have other causes. Additionally, the BLUE procedure (Lichtenstein, 2015), which is commonly used by doctors, does not validate the diagnosis of PTX based just on the absence of lung sliding; other diagnostic techniques, such as lung point detection, are required to validate the diagnosis. Thus, the binary classification of seashore/barcode signs in M-mode images, as well as the classification of visceral and parietal pleural motion in B-mode videos, is not pneumothorax detection, but rather a test for the presence or absence of lung-sliding motion (Jaščur et al., 2021).

Currently, the authors’ known ultrasound pneumothorax studies primarily determine the presence of PTX by detecting the presence or absence of lung sliding, whereas our team, after discussion, concluded that using the absence of lung sliding as the sole basis for determining the positivity of PTX was insufficient. Therefore, after conducting the research and discussion, we attempted to introduce video understanding technology and applied it for the first time in the detection of pneumothorax through ultrasound. By learning the sliding characteristics of the lungs in pneumothorax ultrasound and other related features, we were able to classify the ultrasound video clips of pneumothorax and non-pneumothorax, thereby achieving the goal of pneumothorax detection. Subsequent experiments have proved that this cross-domain application has certain research value in achieving multi-feature detection of medical images.

## 2 Methods and materials

### 2.1 Selection of PTX diagnostic features

**FIGURE 1:**
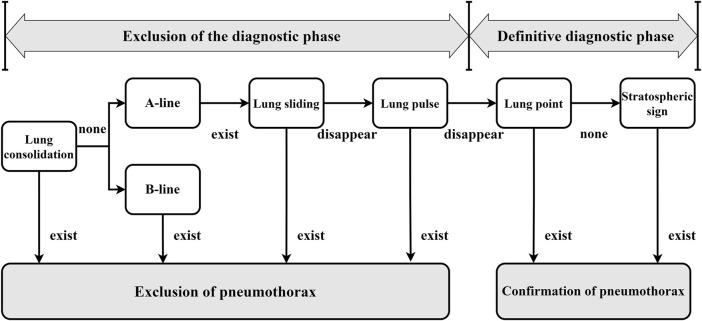
General flow of pneumothorax diagnosis by lung ultrasound (In the clinic, ultrasound pneumothorax characterization is usually performed following the steps and sequence in the figure to rule out or confirm the diagnosis. All of the diagnostic features appearing in the diagnostic process are images under B-mode ultrasound, except for the stratospheric sign, which is an M-mode ultrasound image).

To accurately diagnose lung ultrasound pneumothorax, our team initially investigated the ultrasound pneumothorax diagnostic procedure. The process of diagnosing lung ultrasound pneumothorax (Zhao et al., 2023a) is divided into two stages. ①Diagnostic exclusion stage: Sweep both lungs one by one at each intercostal gap, check ultrasonography signs in the order of “solid lung lesion → B-line → pleural sliding sign → pleural pulsation sign,” and exclude pneumothorax if one of the signs is present. ② Definitive diagnosis stage: If none of the above four indicators exist, use the “lung point sign → stratospheric sign” in order to check for the presence of one or both signals, which can indicate pneumothorax. Refer to Figure 1 for the specific approach.

The diagnostic accuracy of the lung ultrasound pneumothorax diagnostic procedure for PTX was 99.1%, sensitivity was 97.8%, and specificity was 100.0% (Zhao et al., 2023b). There is no pleural slide or pulsation symptom because PTX occurs in only one layer of wall pleura. As a result, the pleural pulsation sign is an essential sign in ruling out PTX and is classified as grade A evidence in the lung ultrasonography evidence-based guidelines (Zhao et al., 2023b). In this study, we feel that when a pleural sliding sign or a pleural pulsation sign is present, we can rule out the potential of a positive PTX and identify it as a negative PTX. As a result, we chose pleural sliding and pleural pulsation signs as crucial criteria for diagnosing negative pneumothorax. When pleural sliding and pulsation disappeared, as well as the existence of lung points or other signs (in clinically verified pneumothorax-positive patients), we used the aforementioned characteristics to diagnose a positive pneumothorax.

### 2.2 Database establishment

This study’s database was created using real clinical data. We formed a collaboration with two hospitals to collect video data from lung ultrasound cases (ultrasound video images from convex and line array probes) accumulated over the previous few years, and all positive and negative instances have been clinically identified and confirmed. We rigorously filtered these clinical data using pre-selected diagnostic features of pneumothorax-negative and pneumothorax-positive to retrieve genuine data that satisfied the study requirements. Given the limited number of clinical cases, we used a data segmentation approach to increase the size of the dataset.

A healthy adult breathes approximately 12–20 times per minute (Wan and Chen, 2015). As a result, we can estimate that each breath lasts three to 5 seconds. Based on this concept, when processing lung ultrasound video clips, we used a 5-second sliding window for segmentation with a 3-second time interval to trim numerous 5-second video clips. As a result, each video segment used for deep learning network training is 5 s long and can ensure that at least one breathing cycle is included in each data segment, allowing for pneumothorax diagnosis. Because the time interval is set to 3 s, there is a 2-second overlap between the two adjacent video clips before and after, but their contents are not exactly the same. This strategy decreases the risk of overfitting while maximizing training data, effectively increasing the amount of data available to us.

By segmenting the raw video data, we were able to collect additional training samples, which improved the model’s training and diagnosis accuracy. After the data segmentation was done, we cleansed the segmented data again to verify its usability and dependability in the research results. We invited two specialized sonographers with more than 8 years of experience in the field to independently screen and clean the data (one with 12 years of experience). They carefully analyzed each segmented video clip to confirm that both positive and negative PTX data utilized for training had acceptable diagnostic features, and they eliminated 17 positive invalid clips and 16 negative invalid clips. Following these data processing processes, we were able to create a dataset with 188 positive clips and 633 negative clips.

**FIGURE 2:**
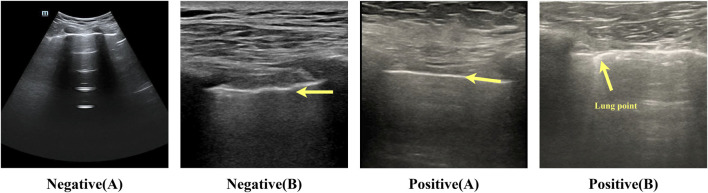
Example of negative-positive data. Negative (A): lung sliding feature, white pleural lines that slide regularly with respiration. The bat sign is a normal ultrasound manifestation in the lungs. Negative (B): lung pulse feature, bright white pleural lines indicated by the arrows in the figure, appear as wavy lines in response to the heartbeat. Positive (A): no lung sliding lung booting feature; bright white pleural lines indicated by the arrows in the figure are straight and non-displaced or in a relatively static state. Positive (B): lung pointing feature; pleural lines appear as discontinuous breakpoints, half of which are sliding and generally not sliding.

**FIGURE 3:**
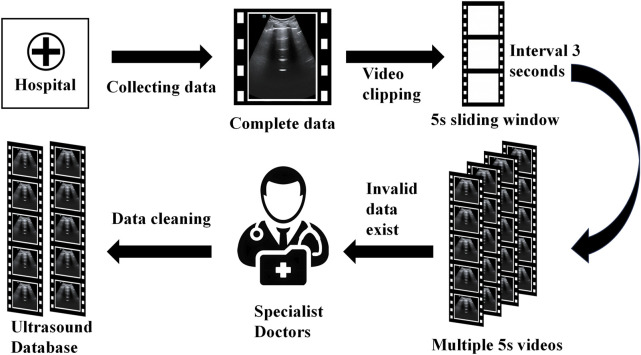
Data Processing Procedure (See the database establishment section for the detailed process).

To ensure the model’s training efficacy and generalization capacity, we partition the dataset into training, validation, and test sets in a 6:2:2 ratio for further model training and evaluation. This type of data split method allows the model to learn a wider range of characteristics during the training process while also testing its performance on the validation and test sets. Table 1 lists information about the data set. Figure 2 shows many examples of data. Figure 3 details the unique data processing flow, clearly showing each step from data collection to data cleaning to data segmentation.

**TABLE 1:** Data Information. The table contains information about the dataset such as the number of datasets, the division ratio of the training validation test data, the features corresponding to the positive data and their number (Multiple features may exist for the same instance of data), the features corresponding to the negative data and their number (Multiple features may exist for the same instance of data) as well as the image resolution, the type of ultrasound probe used, the frame rate of the video, and the device information.

Clips label	Class	Training data (Train and Val)		Holdout data (Test)	
Number of clips	Negative	379 (train)	127 (val)	127(Test)	
	Positive	113 (Train)	38(Val)	37(Test)	
Number of features (There is a crossover of features)	Negative	B-line	Lung sliding	Lung pulse	Lung consolidation
		22	573	70	41
	Positive	A-line	Lung point	No Lung sliding or pulse	No lung consolidation
		136	53	161	1
Duration of data clips	All data segments have a duration of 5 s				
Total frames per clip	MAX: 62 frames * 5 s; MIN: 16 frames * 5 s				
Resolution (of a frame)	800*600; 1,200*900; 1960*910				
Transducers	Curved and Linear				
Machine Vendors	Mindray				

### 2.3 Network structure

Over the years, deep learning has become the norm for video comprehension (Tran et al., 2015; Wang et al., 2016; Carreira and Zisserman, 2017; Wang et al., 2018; Zolfaghari et al., 2018; Xie et al., 2018; Zhou et al., 2018). One important difference between image and video recognition is the need for temporal modeling. For example, altering the order of opening and shutting a box yields the opposite effect, highlighting the importance of temporal modeling (Lin et al., 2019). TSM (Temporal Shift Module) is a generalized and effective time-shift module that may be based on an existing network model to incorporate the temporal shift module technique (the base model used in this paper is ResNet-50, and ResNet-50 is utilized by default in all subsequent sections). It has the performance of a 3D CNN while keeping the intricacy of a 2D CNN. Information can move across adjacent frames thanks to TSM’s transmission of a piece of the channel along the time dimension. For temporal modeling, it may be added to a 2D CNN with no parameters and no processing (Lin et al., 2019).

**FIGURE 4:**
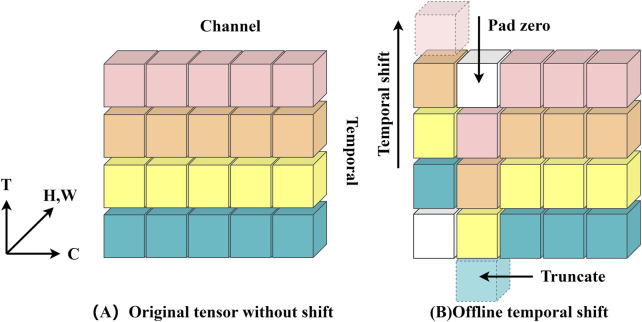
**(A)** depicts the original feature without time shifting; **(B)** depicts the bidirectional time shifting operation (also known as offline time shifting).

The video model’s activation is written as $A\in R^{N\times C\times T\times H\times W}$ , where N is the batch size, C is the number of channels, T is the temporal dimension, and H and W are the spatial resolutions (Lin et al., 2019). Conventional 2D CNNs function independently in the T dimension and so do not exhibit temporal modeling effects, as shown in Figure 4A. The Time Shift Module (TSM), on the other hand, alternately transfers the channels along the time dimension. Following time-shifting, the information from nearby frames is combined with the information from the present frame, as seen in Figure 4B (Lin et al., 2019). To represent offline video recognition, a bidirectional TSM is used. Given a video V, the first T frames Fi, F1,… FT are sampled. After sampling the frames, the 2D CNN baseline processes each frame separately before averaging the logarithm of the outputs to generate the final prediction.

**FIGURE 5:**
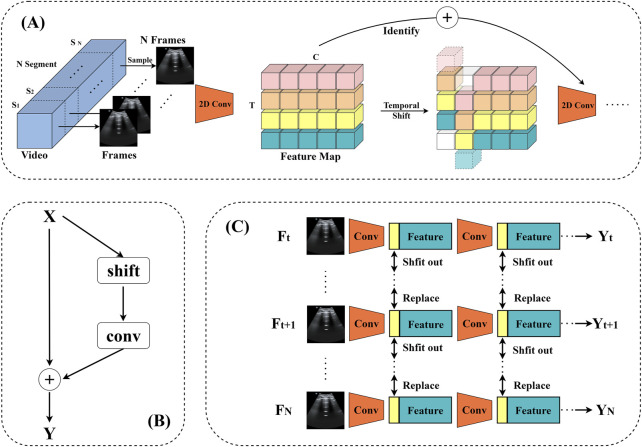
TSM video recognition model. **(A)** The structure of the model. **(B)** TSM residual shift: in order to tackle the degraded spatial feature learning problem, the TSM is positioned within the residual branch of the residual block. This ensures that, following temporal shifting by constant mapping, all of the information from the initial activation is still available. **(C)** Bidirectional TSM inference map for video identification. In order to construct the next layer of features, the first 1/8 feature maps of each residual block are retained for each frame throughout the inference phase. These are then substituted with the frames that came before and after.

The model’s data input consists of a batch of ten RGB images with a tensor form of (10, 3, 224, 224), indicating that each image is 224 × 224 pixels with three color channels. Following a series of convolution, batch normalization, ReLU activation, and maximum pooling, the data successfully passes through the initial feature extraction stage and enters the first residual block, where it is reshaped to (10, 64, 56, 56). The information fusion between frames is then realized by performing “right shift” and “left shift” operations on this tensor, which replace the corresponding part of the first nine frames with one-eighth of the data chosen from each channel of the last nine frames, and then replacing the corresponding part of the last nine frames with one-eighth of the data of the first nine frames. This tactic creates the impression that “you are in me, I am in you” and improves the information exchange across frames. The residual block is then used to learn from the tensor following the shift. Notably, the model’s sensory field doubles with each embedding of the time-shift module in the temporal dimension, much like when a convolution with a kernel size of three is applied to the time series. Figure 5 illustrates the model’s structure. Therefore, the model using TSM has a broad spatio-temporal sensory field and is capable of modeling fine spatio-temporal relationships, which is an extremely effective technological tool for video analytics and other tasks that require simultaneous consideration of spatial and temporal dimensions.

### 2.4 Model training framework

**FIGURE 6:**
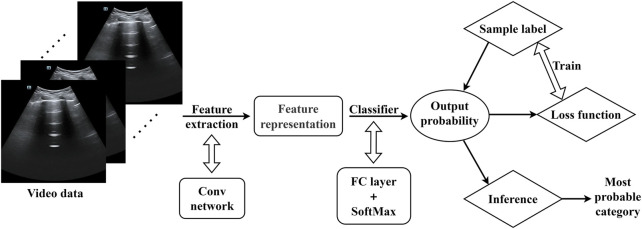
Model training framework (The video data were randomly scaled, cropped, and flipped before being entered into the model).

The training structure is depicted in Figure 6. The model used in the code is a TSM network structure based on a single RGB picture, and the backbone network is the traditional ResNet-50 design. Each training video is divided into 10 segments (each lasting 0.5 s) during the data preparation step. One frame is randomly selected from each segment (10 frames total), and the data is then fed into the training model following uniform processing (random scaling, cropping, flipping). The model initially extracts features from the input ultrasound image data using a convolutional neural network, which yields a rich feature representation. These features are then fed into a classifier, which calculates the probability distribution of each video feature class. A loss function is built during training based on the discrepancy between the model’s output probability values and the real sample labels, and the model is then optimized. In order to reduce the discrepancy between the expected and actual values, this stage is essential to model learning. In the inference stage, the model will produce the final prediction for the category with the highest probability, ensuring that ultrasound image data is accurately classified.

### 2.5 Training configuration and parameters

Training was performed on a server configured with Ubuntu 16.04 operating system, using a single GPU, model TITAN RTX. Model construction was based on the PaddlePaddle 2.6 framework. In order to improve the starting performance of the model, we used the ResNet-50 model weights pre-trained on the ImageNet dataset as initialization parameters for the backbone network.

We established the following parameters for the dataset’s training configuration: A total of 100 training cycles (epochs) were carried out, with eight samples in each batch (batch size). To avoid overfitting, the dropout ratio was set to 0.8, and the optimizer’s momentum parameter was set to 0.9. With an initial learning rate of 0.001, the decay strategy for the learning rate (LR) was set up as follows. It was carried out on a boundary of 10–20 epochs, and anytime the decay boundary was reached, the learning rate was decreased to 0.1 times its initial value. Additionally, we froze the Batch Normalization (BN) layer (Zolfaghari et al., 2018) and adjusted the pre-trained weights from ImageNet throughout model training to ensure stability.

## 3 Result

### 3.1 Result of training

**FIGURE 7:**
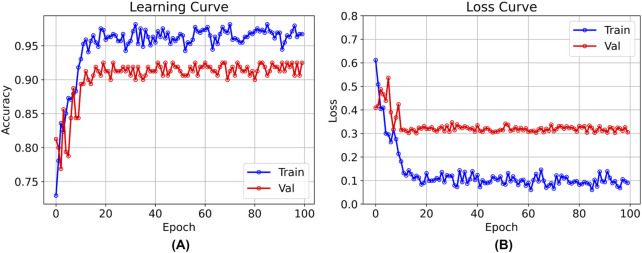
The training and validation curves after 100 epochs of training using the TSM-ResNet50 model [**(A)** shows the process of accuracy change, and **(B)** shows the process of loss value change].

**FIGURE 8:**
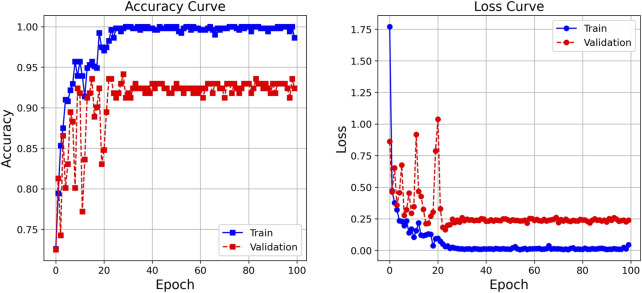
Training and validation curves of the ResNet-50 model for 100 epochs.

**FIGURE 9:**
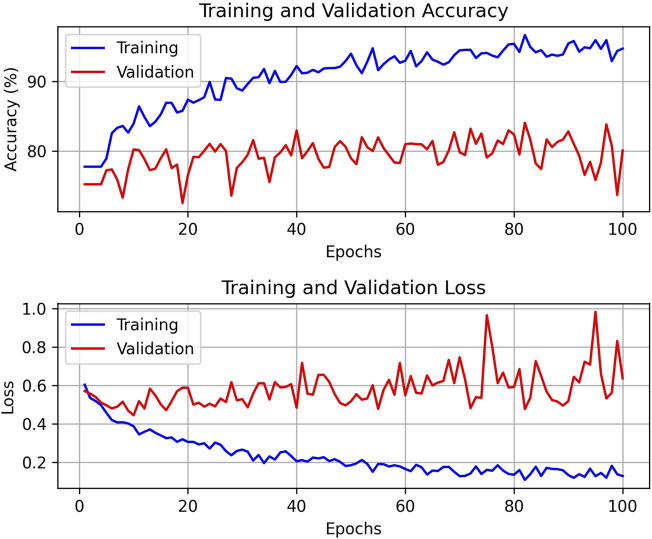
The training curve and validation curve of the CNN-LSTM model over 100 cycles.

After 100 rounds of training using the above training parameter settings, the learning curve of the TSM-ResNet50 model is shown in Figure 7A and the loss curve is shown in Figure 7B. The training accuracy of the model is up to 98.15% and the model also achieves 92.5% accuracy on the validation set. The value of the loss value for the training of the model stabilized around 0.1, and the value of the loss for the validation set stabilized around 0.3, and from the training metrics, the model learned the features we picked as we expected it to. For comparison, the same dataset was used to train the ResNet-50 model without adding the TSM module and the CNN-LSTM model, and the tests were conducted on the same test set. The training curve of the ResNet-50 model is shown in Figure 8, and its highest accuracy on the validation set was 94.15%. The training curve of the CNN-LSTM model is shown in Figure 9, and its highest accuracy on the validation set was 86.03%.

### 3.2 Result of test

**FIGURE 10:**
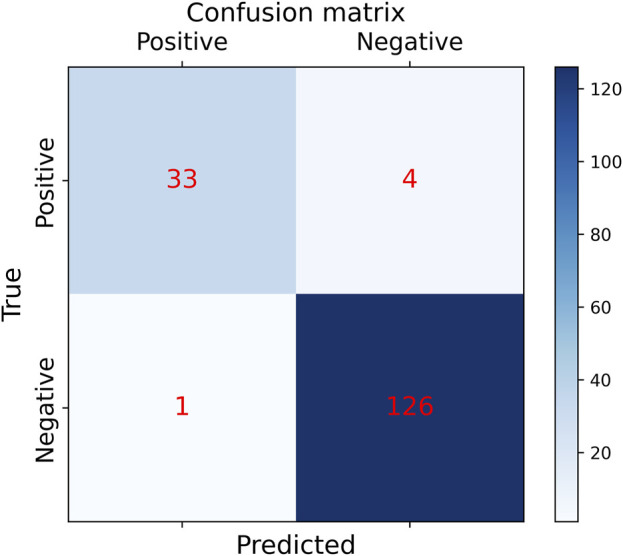
Results of the confusion matrix for prediction on the test set using the ResNet-50 model with the TSM module (final results of the test set classification, TP=33, TN=126, FP=1, FN =4).

**FIGURE 11:**
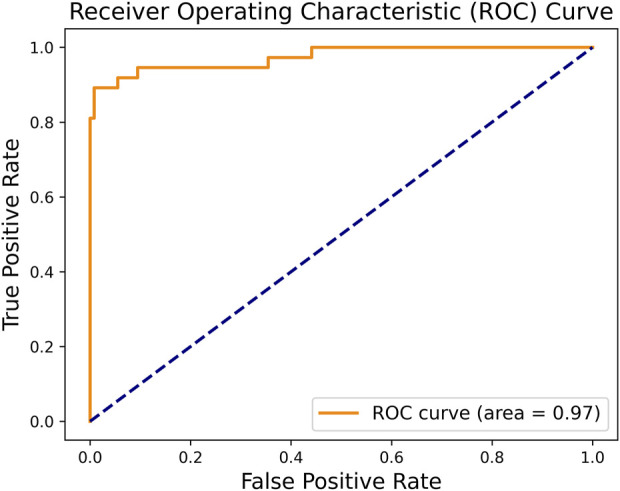
By making predictions on the test set and obtaining the prediction probabilities, the ROC curve of the TSM-ResNet50 model was plotted.

**FIGURE 12:**
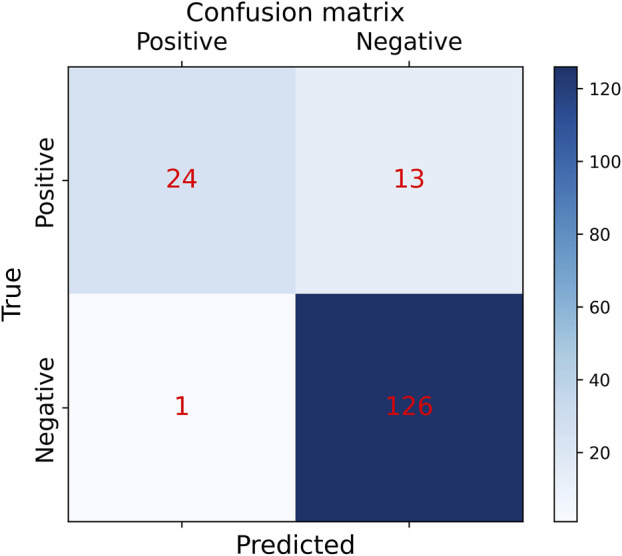
Confusion matrix results of making predictions on the test set using the ResNet-50 model (final results of the test set classification, TP=24, TN=126, FP=1, FN =13).

**FIGURE 13:**
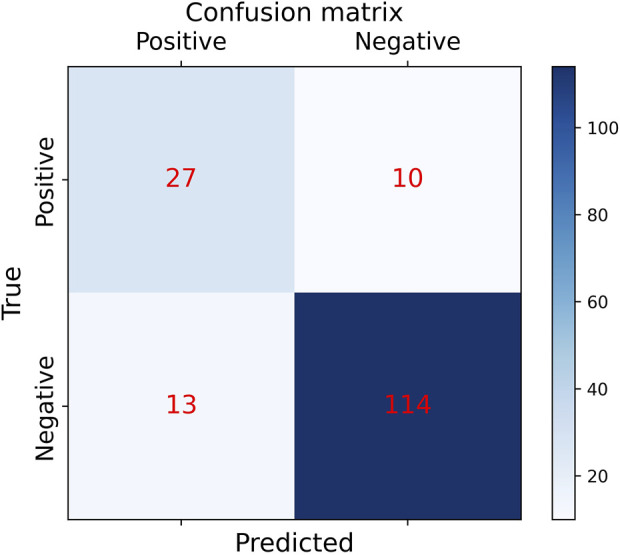
Confusion matrix results of making predictions on the test set using the CNN-LSTM model (final results of the test set classification, TP=27, TN=114, FP=13, FN =10).

In order to the actual performance of the TSM-ResNet50 model and to evaluate it, we tested the trained model using 164 cases of data (37 positive and 127 negative) from the test set (data not involved in the training) that was kept before the training. Of the 164 cases of test data, 159 cases of data were correctly predicted, 33 cases were positive and 126 cases were negative. There were 5 cases of incorrectly predicted data, one negative and four positive cases, and the model achieved an overall recognition accuracy of 96.95%, a precision (check rate) of 97.06%, a recall (sensitivity) of 89.18%, a specificity of 99.21%, and an F1_score of 0.9692, the confusion matrix of the TSM-ResNet50 model is shown in Figure 10, and the ROC curve is shown in Figure 11. The confusion matrix of the ResNet-50 model without using the TSM module on this test set is shown in Figure 12, while the confusion matrix of the CNN-LSTM model on this test set is shown in Figure 13. The test performance of all the trained models on the independent test set is listed in Table 2. Taking all these evaluation indicators into consideration, the test results of the TSM-ResNet50 model are all superior to those of the ResNet-50 model and the CNN-LSTM model.

**TABLE 2:** The test performance of all models on the same test set.

Indicator Model	TSM- ResNet50	ResNet-50	CNN- LSTM
Accuracy	0.9695	0.9146	0.8597
Precision	0.9706	0.9600	0.6750
Recall	0.8919	0.6486	0.7297
Specificity	0.9921	0.9921	0.8976
F1_score	0.9296	0.7742	0.7013
Model parameter	94,289,074	94,367,681	192,999,414

### 3.3 Misanalysis

**FIGURE 14:**
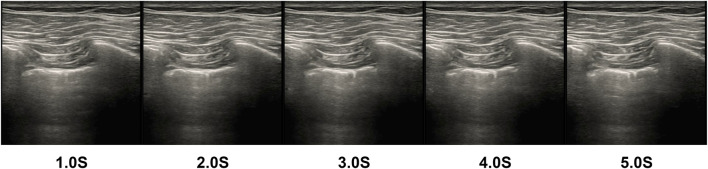
Cases predicted as positive by the model in negative data (Picture frames from the ultrasound video were intercepted at 1-second intervals and stitched together into 1- to 5-second screenshots, with the bright white pleural line in the center of each image almost at rest).

In the test, there was one case where negative data was predicted to be positive. Our model considered the likelihood of the data being negative to be 29.63% and the likelihood of the data being positive to be 70.37%, so the data was labeled as positive, and we extracted and analyzed the data from that case. The image of the example data is shown in Figure 14. The feature of this data that was labeled as negative by the expert was the presence of lung sliding, and we found that there was indeed lung sliding in this data after reviewing the raw data, but the lung sliding appeared between 3.5 and 5 s, and the pleural line was almost at a standstill before 3.5 s, which was a large percentage of the positive features and an inconspicuous percentage of the negative features, which led to the prediction error.

**FIGURE 15:**
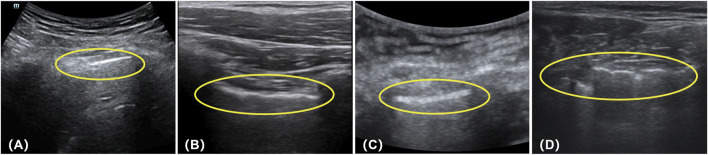
The data of **(A–D)** in the figure are all positive, but the model previously predicted their results as negative (the circled parts in the figure represent the pleural lines present in each image).

There were four cases of positive data in the test that were predicted to be negative, and we also analyzed them separately. Figure 15A, positive data labeled by the expert as no lung sliding and no pleural pulsation but predicted by our model to be negative with a 94.47% probability, was analyzed to show that the pleural line in the picture did not undergo lung sliding but produced a large movement (a movement similar to lung sliding), which resulted in the model incorrectly judging the movement of the pleural line to be lung sliding. Figure 15B, again positive data without lung sliding and lung pulsation, but the pleural line produced a wavy line of pulsation (very similar to the pleural pulsation sign), and the presence of pleural pulsation was negative, so the model incorrectly judged it as negative. Figure 15C, the expert gave this data the classification feature of A line, but the pleural line in the picture is too blurred, the contrast with other lung tissues is low, there is a large movement in the picture, and the model did not accurately recognize the diagnostic feature and misjudged it as negative. Figure 15D, the data situation of this case is similar to that in Figure 15A, the pleural line in the picture showed a large degree of lateral movement, which was recognized as negative by the model. By analyzing the data of the above prediction errors, the following conclusion can be deduced: when the position of the pleural line in the positive data frame is shifted to a large extent, it is easy for the model to misdiagnose. The reason for this may be that the weight of positive data is small compared to negative data, the weight of negative features is too high, and the model is not fine enough to recognize the positive features.

## 4 Discussion

Interpretation of medical images is often performed by professionals, and compared to other medical images, ultrasound images are more difficult to interpret. Ultrasound plays an important role in the initial screening of medical conditions. Therefore, if the diagnostic threshold of ultrasound can be lowered, ultrasound technology can play a greater role in more fields, and in the future, ultrasound can be operated remotely in real-time or unmanned automated diagnosis. Our work is based on the above objectives and focuses on the intelligent diagnosis of ultrasound pneumothorax.

In this report, we propose a technical solution for accurately recognizing ultrasound pneumothorax, a first attempt to directly use video comprehension techniques for ultrasound pneumothorax diagnosis, and the results of the study show that AI models also have an extremely great potential for the interpretation of ultrasound images. Through deep learning algorithms, the model is able to learn the abstract features of PTX in ultrasound images, thus realizing a more accurate diagnosis. This finding is of great significance in lowering the threshold of ultrasound image diagnosis, especially in scenarios where medical resources are limited. The AI-assisted diagnosis system is expected to improve the diagnostic capability of primary care organizations.

From the current point of view, deep learning research in ultrasound diagnostic PTX still lags behind more traditional chest imaging techniques such as CT or X-ray, in which organized and labeled data are easier to acquire (Thian et al., 2021; Taylor et al., 2018; Röhrich et al., 2020) while lung ultrasound data acquisition is still difficult. Although some scholars have begun to engage in research related to lung ultrasound pneumothorax, they have adopted much the same approach, starting from B-mode ultrasound and focusing on the interpretation of lung sliding of M-mode images after reconstruction of B-mode ultrasound images into pseudo-M-mode images (VanBerlo et al., 2022; Boice et al., 2022; Jaščur et al., 2021). The difference is what kind of model or strategy is used to reconstruct and categorize M-mode images from B-mode ultrasound. Our work starts from another angle, applying the video understanding technology in the field of ultrasound diagnosis, directly classifying and diagnosing the B-mode ultrasound images, just like a doctor who has been practicing medicine for many years, directly reading the ultrasound images without the need of reconstructing M-mode ultrasound images, and our experimental results show that this technical route is feasible. With the advantage of this technique, we can save a lot of time and labor costs in data annotation, and this advantage will be more obvious in large databases. VanBerlo et al. believe that it is better to include pleural pulsation sign in the detection of PTX (VanBerlo et al., 2022), but their study focuses on a single judgment of pleural sliding like previous studies. Our work not only focuses on pleural sliding but also adds some other key features used to diagnose pneumothorax, such as pleural pulsation sign, lung points (Zhao et al., 2023a), and so on. The diagnosis of ultrasound pneumothorax by fusion of multiple features has been realized using video comprehension techniques, and good results have been achieved. By learning the fusion of multiple key features in the diagnostic process of ultrasound pneumothorax, the positive and negative abstract features in the ultrasound pneumothorax video are directly categorized, which further improves the accuracy of pneumothorax diagnosis.

Extrapolating from the existing findings of our current work, as well as the research results related to video understanding in the field of natural imagery, theoretically, if we have a large enough variety of cases in our database and a large enough amount of image data, we can perform the diagnostic classification work of multiple diseases at the same time, and the diagnostic accuracy will be further improved, and this research will become even more meaningful. However, as with other medical models, the lack of interpretability of the models is an issue that needs to be addressed to ensure that clinicians can understand and trust the diagnostic results of the models. Therefore, currently AI models are only used as auxiliary tools to help doctors do their jobs more efficiently. How to combine AI models with doctors’ expertise to achieve human-machine collaboration is also another important direction for future research. However, the amount of data available for training is still limited by the size of the dataset. As a result, we think that future studies will concentrate on growing the database, including additional disease categories, enhancing model performance, and resolving the model’s interpretability. Despite the fact that the diagnosis of ultrasonic pneumothorax was the main focus of our work, the model and technique that were developed may have wider uses. In the future, we can use this method to diagnose additional ultrasound images, like liver and heart diseases, expanding the applications of ultrasound technology and giving patients faster, more precise medical care.

## 5 Conclusion

In conclusion, the development of a straightforward and user-friendly ultrasound image acquisition and diagnosis system will tremendously aid general medical staff in the pre-hospital emergency stage for prompt diagnosis, hence increasing patient survival rates. Ultrasound technology’s ease of use and effectiveness in emergency medical settings will guarantee that patients can be quickly evaluated, screened, and triaged, improving medical institutions’ emergency response capabilities. The current study is a component of the intelligent diagnostic system called E-FAST (Extended Focused Assessment with Sonography for Trauma), which is designed to determine whether a patient has a PTX. Looking ahead, our next plan is to optimize the training strategy and expand the database in order to build diagnostic models with more generalization ability for diagnosis of a wide range of diseases. To give users a more practical and effective diagnostic experience, we will also investigate integration with portable handheld ultrasound instruments. We anticipate that these initiatives will transform the healthcare industry and advance the adoption of diagnostic ultrasonography technology.

## Data Availability

The original contributions presented in the study are included in the article/Supplementary Material, further inquiries can be directed to the corresponding author/s.
