# Supplementary material for: Use video comprehension technology to diagnose ultrasound pneumothorax like a doctor would
Source: Front Physiol. 2025 May 27;16:1530808. doi: 10.3389/fphys.2025.1530808 (PMC12148891; doi:10.3389/fphys.2025.1530808)
Supplement: Supplementary file 1 [file DataSheet2.pdf]

Supplementary Material

1.1 Supplementary Figures

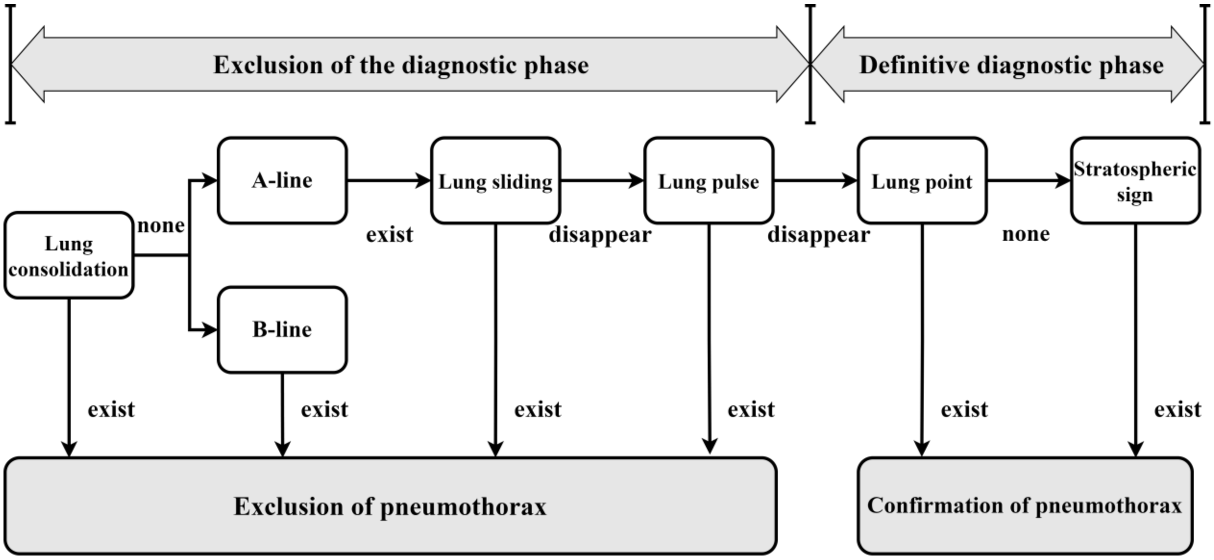

Supplementary Figure 1. General flow of pneumothorax diagnosis by lung ultrasound (In the clinic, ultrasound pneumothorax characterization is usually performed following the steps and sequence in the figure to rule out or confirm the diagnosis. All of the diagnostic features appearing in the diagnostic process are images under B-mode ultrasound, except for the stratospheric sign, which is an M-mode ultrasound image.).

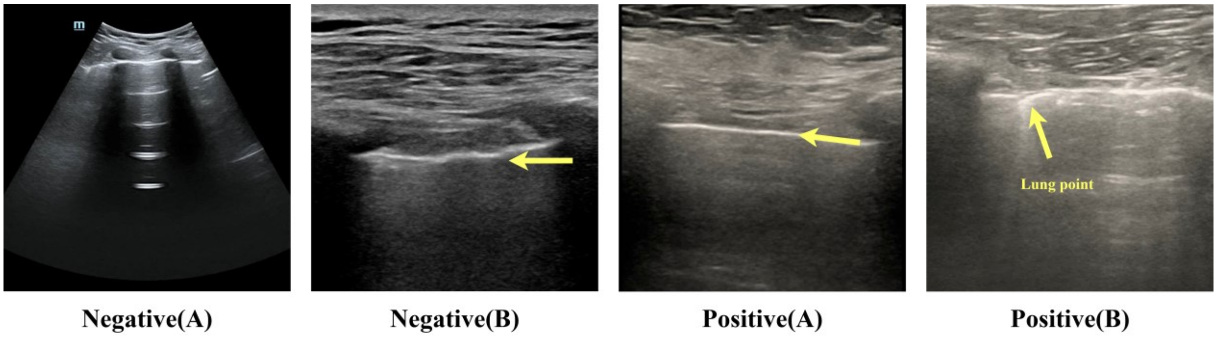

Supplementary Figure 2. Example of negative-positive data. Negative (A): lung sliding feature, white pleural lines that slide regularly with respiration. The bat sign is a normal ultrasound manifestation in the lungs. Negative (B): lung pulse feature, bright white pleural lines indicated by the arrows in the figure, appear as wavy lines in response to the heartbeat. Positive (A): no lung sliding lung booting feature; bright white pleural lines indicated by the arrows in the figure are

straight and non-displaced or in a relatively static state. Positive (B): lung pointing feature; pleural lines appear as discontinuous breakpoints, half of which are sliding and generally not sliding.

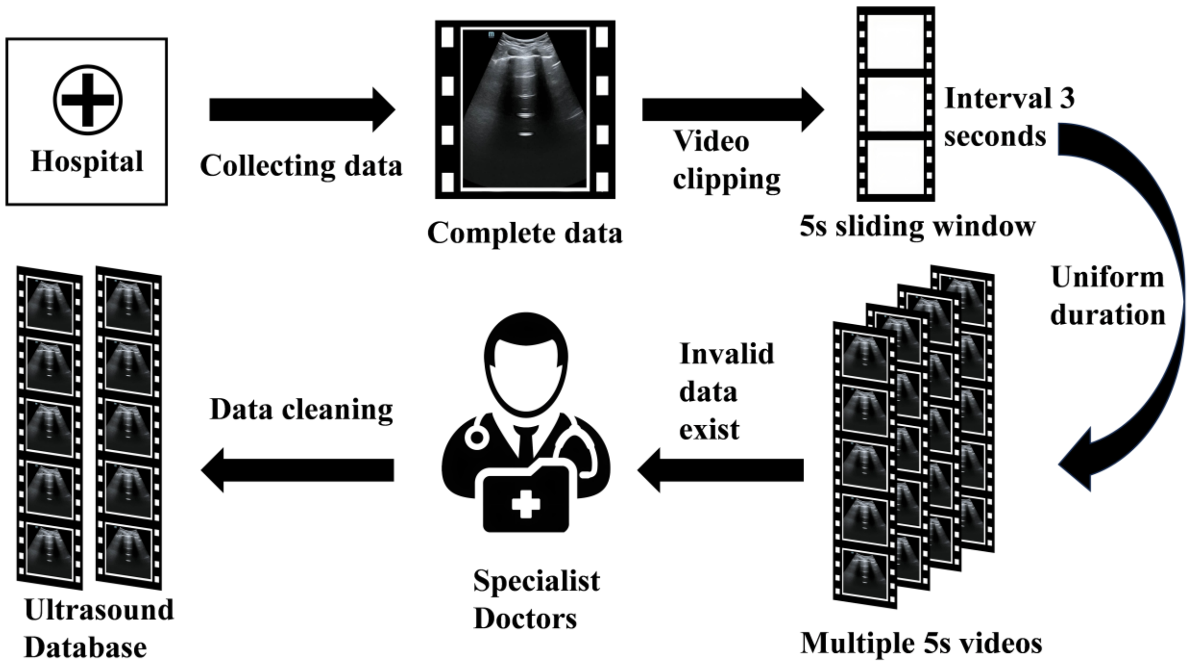

Supplementary Figure 3. Data Processing Procedure (See the database establishment section for the detailed process).

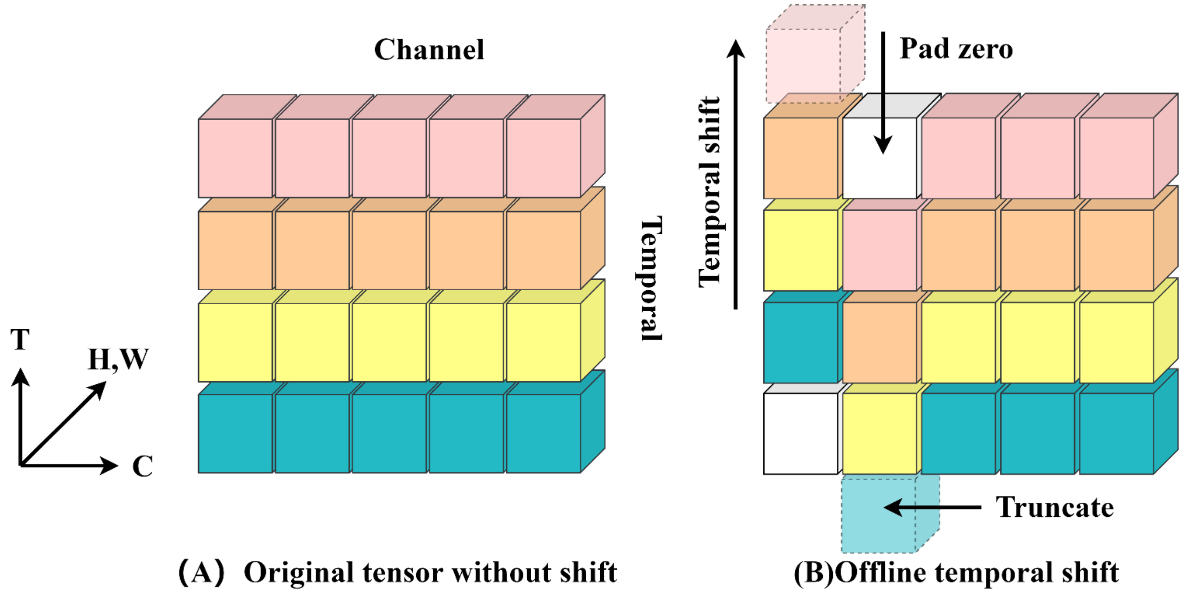

Supplementary Figure 4. (A) depicts the original feature without time shifting; (B) depicts the bidirectional time shifting operation (also known as offline time shifting).

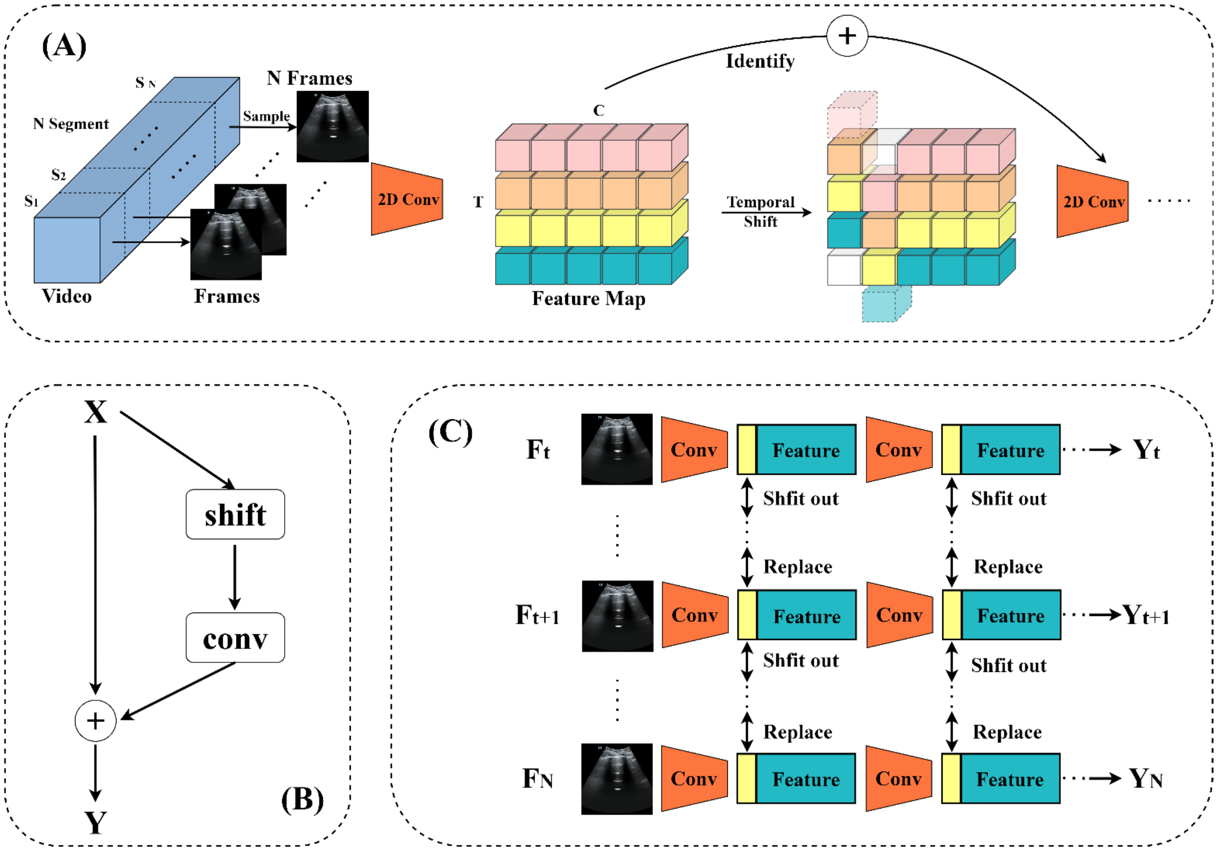

Supplementary Figure 5. TSM video recognition model. (A) The structure of the model. (B) TSM residual shift: in order to tackle the degraded spatial feature learning problem, the TSM is positioned within the residual branch of the residual block. This ensures that, following temporal shifting by constant mapping, all of the information from the initial activation is still available. (C) Bidirectional TSM inference map for video identification. In order to construct the next layer of features, the first

1/8 feature maps of each residual block are retained for each frame throughout the inference phase. These are then substituted with the frames that came before and after.

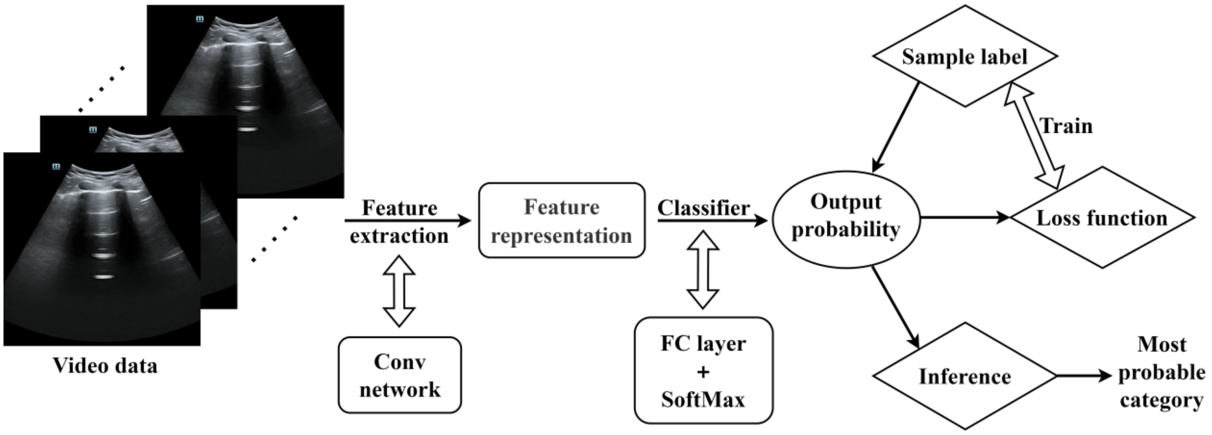

Supplementary Figure 6. Model training framework（The video data were randomly scaled, cropped, and flipped before being entered into the model.）.

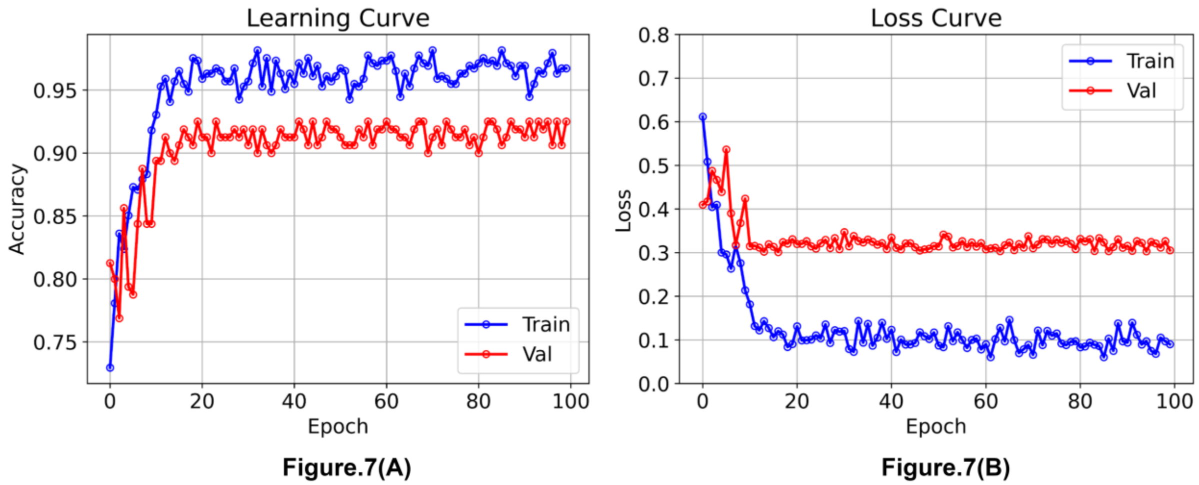

Supplementary Figure 7. Training and validation curves of the Resnet-50 model with the TSM module for 100 epochs.

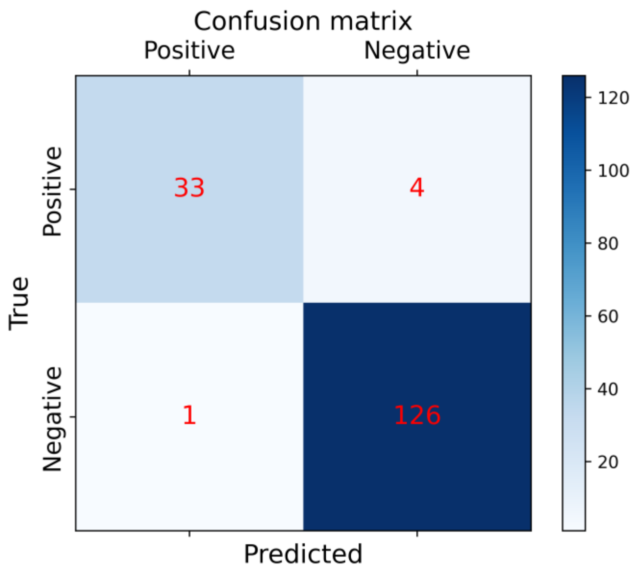

Supplementary Figure 8. Results of the confusion matrix for prediction on the test set using the Resnet-50 model with the TSM module (final results of the test set classification, TP=33, TN=126, FP=1, FN =4).

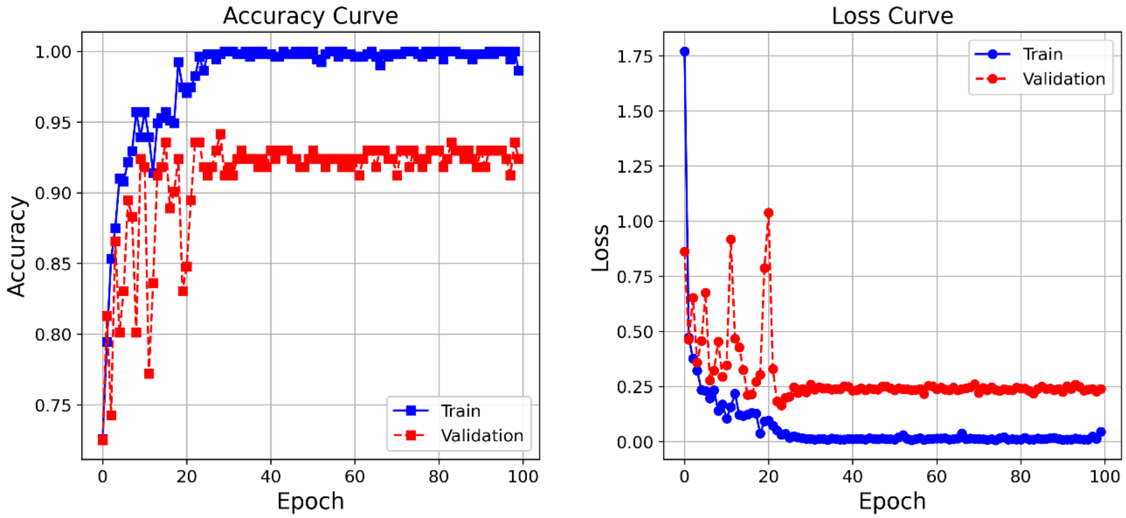

Supplementary Figure 9. Training and validation curves of the Resnet-50 modelfor 100 epochs.

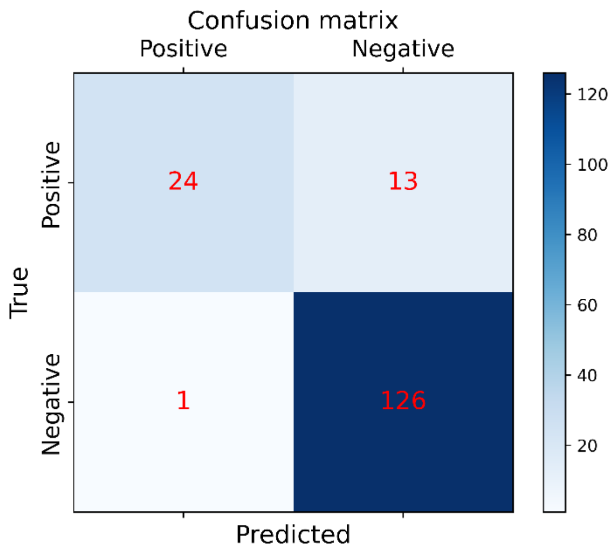

Supplementary Figure 10. Results of the confusion matrix for prediction on the test set using the Resnet-50 model with the TSM module (final results of the test set classification, TP=24, TN=126, FP=1, FN =13).

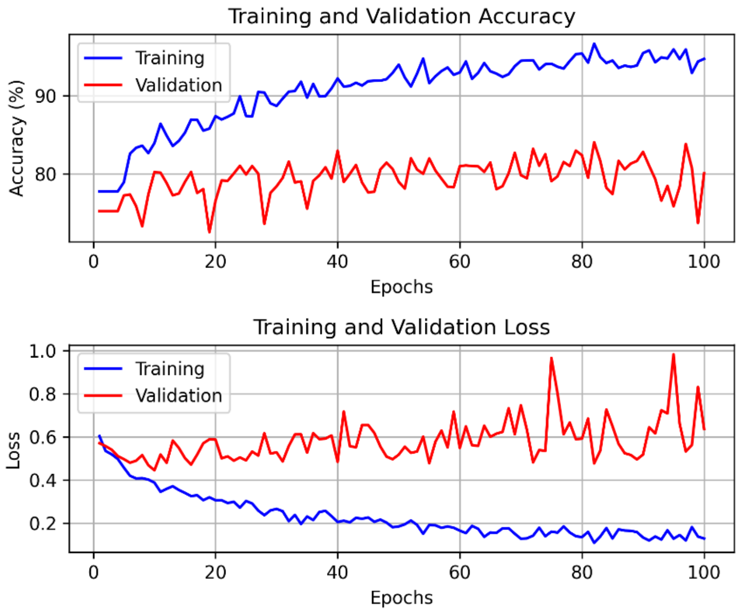

Supplementary Figure 11. The training curve and validation curve of the CNN-LSTM model over 100 cycles.

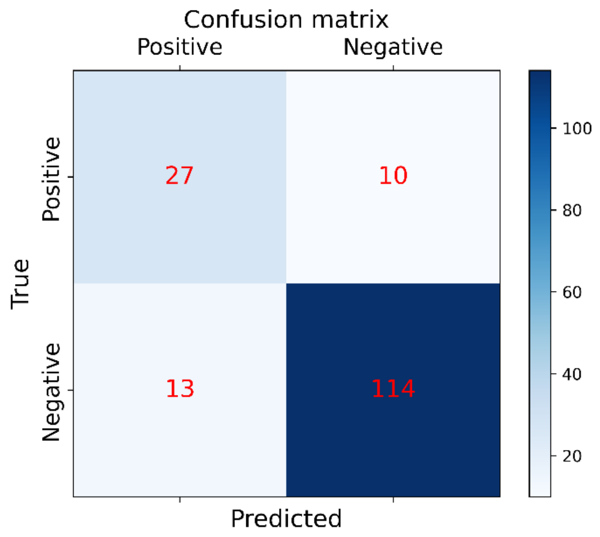

Supplementary Figure 12. Results of the confusion matrix for prediction on the test set using the Resnet-50 model with the TSM module (final results of the test set classification, TP=27, TN=114, FP=13, FN =10).

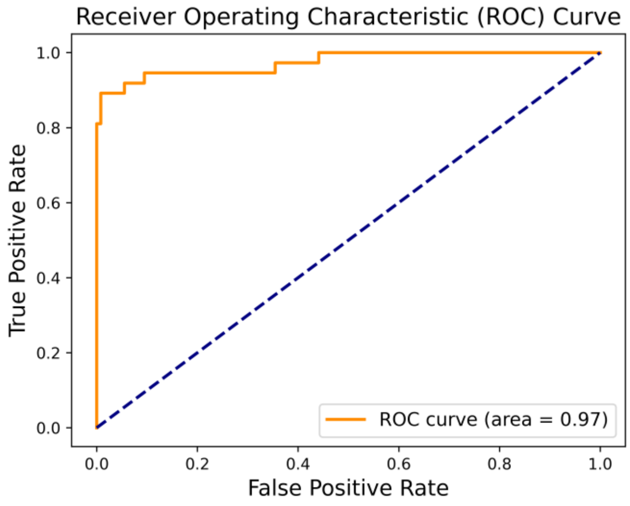

Supplementary Figure 13. By making predictions on the test set and obtaining the prediction probabilities, the ROC curve of the TSM-Resnet50 model was plotted.

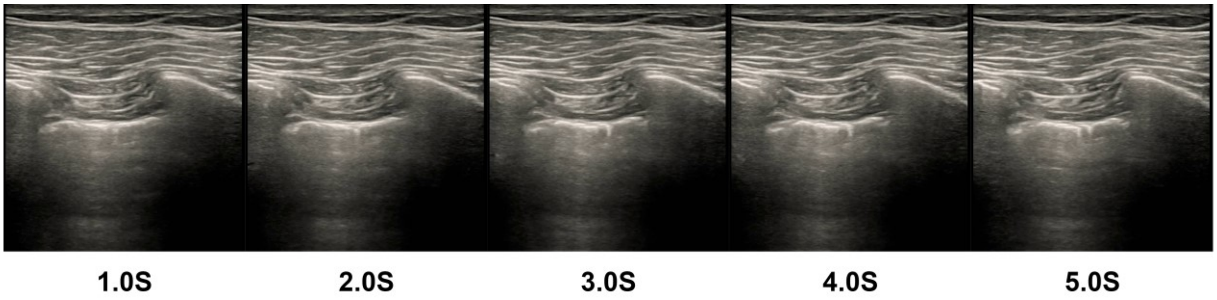

Supplementary Figure 14. Cases predicted as positive by the model in negative data（Picture frames from the ultrasound video were intercepted at 1-second intervals and stitched together into 1- to 5-second screenshots, with the bright white pleural line in the center of each image almost at rest）.

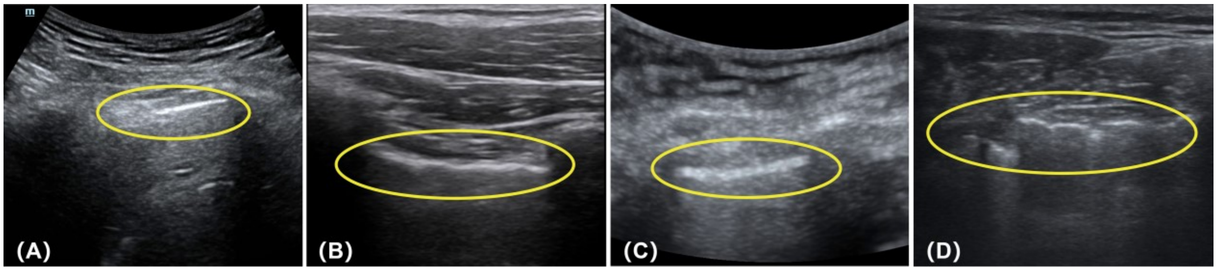

Supplementary Figure 15. Four cases of positive data that were predicted to be negative by the model（Circled in the figure are the pleural lines that appear in each image）
